# Trajectory of adjustment difficulties following disaster: 10-year longitudinal cohort study

**DOI:** 10.1192/bjo.2024.3

**Published:** 2024-03-04

**Authors:** Belinda J. Pacella, Sean Cowlishaw, Lisa Gibbs, Richard A. Bryant, Kate Brady, Colin Gallagher, Robyn Molyneaux, Kari Gibson, Karen Block, Louise Harms, David Forbes, Meaghan L. O'Donnell

**Affiliations:** Phoenix Australia, Centre for Posttraumatic Mental Health, Department of Psychiatry, University of Melbourne, Melbourne, Australia; Turner Institute for Brain and Mental Health, Monash School of Psychological Sciences, Monash University, Melbourne, Australia; Child and Community Wellbeing Unit, Melbourne School of Population and Global Health, University of Melbourne, Melbourne, Australia; School of Psychology, University of New South Wales, Sydney, Australia; Centre for Transformative Innovation, Faculty of Business and Law, Swinburne University of Technology, Melbourne, Australia; Department of Social Work, Melbourne School of Health Sciences, University of Melbourne, Melbourne, Australia

**Keywords:** Disasters, bushfires/wildfire, adjustment disorders, longitudinal, post-disaster mental health

## Abstract

**Background:**

Although much is known about psychopathology such as post-traumatic stress disorder (PTSD) and depression following bushfire (also known as wildfire), little is known about prevalence, trajectory and impacts for those experiencing general adjustment difficulties following exposure to these now-common events.

**Aims:**

This was an exploratory analysis of a large cohort study that examined the prevalence, trajectory and risk factors of probable adjustment disorder over a 10-year period following bushfire exposure.

**Method:**

The Beyond Bushfires study assessed individuals exposed to a large and deadly bushfire across three time points spanning 10 years. Self-report survey data from participants from areas with moderate and high levels of fire-affectedness were analysed: *n* = 802 participants at Wave 1 (3–4 years post-fires), *n* = 596 at Wave 2 (5 years post-fires) and *n* = 436 at Wave 3 (10 years post-fires). Surveys indexed fire-related experiences and post-fire stressors, and comprised the six-item Kessler Psychological Distress Scale (probable adjustment disorder index), four-item Posttraumatic Stress Disorder Checklist (probable fire-related PTSD) and nine-item Patient Health Questionnaire (probable major depressive episode).

**Results:**

Prevalence of probable adjustment disorder was 16% (Wave 1), 15% (Wave 2) and 19% (Wave 3). Probable adjustment disorder at 3–4 years post-fires predicted a five-fold increase in risk for escalating to severe psychiatric disorder (i.e. probable fire-related PTSD/major depressive episode) at 10 years post-fires, and was associated with post-fire income and relationship stressors.

**Conclusions:**

Adjustment difficulties are prevalent post-disaster, many of which are maintained and exacerbated over time, resulting in increased risk for later disorder and adaptation difficulties. Psychosocial interventions supporting survivors with adjustment difficulties may prevent progression to more severe disorder.

Disasters, both natural and man-made, are potentially traumatic events that involve threat of personal harm, loss of life and far-reaching damage to a community's physical, social and economic infrastructure.^1,2^ Twenty years of disaster research attests to the potential mental health effects of these events, demonstrating that although most individuals are resilient and do not develop mental health difficulties in the aftermath of such events, a proportion of individuals experience increased rates of psychological distress and psychiatric disorder.^2,3^ Post-disaster literature has predominantly focused on post-traumatic stress disorder (PTSD), with prevalence rates estimated from 5 to 22%,^4–8^ and major depressive disorder (MDD), with prevalence estimated at 16%.^8^

## Subthreshold symptomatology and adjustment disorder

In addition to those who develop diagnosable mental health conditions, a considerable portion of individuals experience subthreshold symptomatology. Subthreshold symptomatology refers to those whose symptoms do not meet criteria for disorder but who do report high levels of symptoms or distress. Self-reported rates of psychological distress can be as high as 42% around 12 months following bushfire exposure.^9^ Rates of subthreshold PTSD have been found to be double those of full PTSD in the early aftermath of earthquake (28.0%, 13.8% respectively^10^ and 19.0%, 10.3%^11^) and terror attack exposure (15.7%, 4.8%^12^) and in the 12 months following terror attack exposure (17.5%, 9.3%^13^). Although PTSD and MDD are the most frequently studied clinical outcomes in disaster literature,^14^ research suggests that a broad range of subthreshold symptoms may be the most prevalent mental health outcome following disaster.

Examining the occurrence and course of subthreshold symptomology in the aftermath of disaster is important, as such symptoms are associated with many negative psychological outcomes in both trauma-exposed and non-trauma-exposed populations, including reduced quality of life,^15^ increased suicide risk^16^ and social and occupational impairment.^17^ Of critical concern is research that demonstrates subthreshold symptomology to be associated with an equal degree of dysfunction and functional impairment when compared with both MDD^18,19^ and PTSD.^17^ This again speaks to the importance of directing research and clinical attention towards understanding subthreshold symptomology following disaster exposure.

In recognition of the importance of subthreshold psychopathology, DSM-5 has reclassified adjustment disorder to sit alongside PTSD in the ‘Trauma and stressor-related disorders’ chapter. In response to a psychosocial stressor, symptoms of PTSD or major depressive episode (MDE) that do not meet their respective DSM-5 diagnostic thresholds are now directed to adjustment disorder.^20^ Emerging evidence supports this approach, indicating that adjustment disorder may sit on a continuum of symptom severity between no disorder and other psychiatric disorders.^21^

## Post-trauma adjustment disorder research

Despite recognition that adjustment difficulties and subclinical symptoms are an important outcome following trauma, most research exploring adjustment difficulties has not been conducted after trauma. As a result, there is little understanding pertaining to the prevalence, trajectories and risk factors of adjustment difficulties in the aftermath of exposure to potentially traumatic events such as disasters, nor evidence pertaining to effective interventions for those with these difficulties.^22–24^ There is little understanding of what potential risk factors implicate an individual's trajectory towards adjustment difficulties versus a more severe disorder following trauma, although one study found physical proximity to the traumatic event to differentiate a diagnosis of PTSD from adjustment disorder.^25^

In one of the few longitudinal studies to date, O'Donnell and colleagues (2016) examined adjustment disorder in a sample of traumatic injury survivors (i.e. motor vehicle accident, fall, assault), with prevalence rates estimated at 19% 3 months post-injury and 16% 12 months post-injury.^21^ Those with adjustment disorder (relative to no disorder) at 3 months were found to be 2.7 times more likely to meet criteria for a more severe psychiatric disorder (PTSD, MDD or anxiety) at 12 months, evidencing the importance of adjustment disorder as a potential gateway to more severe disorder over time. Further, the majority of trauma survivors who developed adjustment disorder did so after the 3-month post-injury period, contravening the DSM-5 diagnostic criterion, which requires the onset of adjustment disorder to commence within 3 months of the stressor. This finding presents the possibility that individuals may develop adjustment difficulties well after the occurrence of a traumatic event, when the personal, social or financial consequences associated with the event are realised. This is a particular issue in post-disaster contexts, where acute post-disaster stressors (e.g. displacement, property damage) that may have immediate impacts on mental health^26^ are often compounded by longer-term post-disaster stressors (e.g. ongoing financial concerns, relationship conflicts, insurance claim difficulties) that play additional roles in the course of psychological symptoms.^14^ Although exposure to a disaster itself may act as a trigger stressor for the development of adjustment disorder, the ongoing financial, health or interpersonal stressors^27,28^ that ensue in the months following the disaster may play a key role in increasing risk for the persistent course of adjustment disorder.

## The Beyond Bushfires study

In January and February 2009, Australia experienced wide-ranging bushfires, with the most severe occurring on 7 February, subsequently referred to as the Black Saturday bushfires. The bushfires caused widespread damage across the state of Victoria, resulting in 173 fatalities, 3500 buildings damaged, including over 2000 homes, and severe impact on community infrastructure.^29^ The Beyond Bushfires study is a programme of research undertaken by the University of Melbourne in partnership with community, government and emergency agencies to evaluate the longitudinal mental health and community outcomes in regions of Victoria affected by the fires.^30^ The study conducted three waves of data collection, across 10 years following exposure to the fires. Mental health outcomes for Wave 1, Wave 2 and Wave 3 have been reported elsewhere.^31–33^

The aim of the present study was to explore the longitudinal trajectory and risk factors of probable adjustment disorder across the 10 years following exposure to the 2009 Black Saturday bushfires. The term ‘probable’ disorder was used because we used simple, self-reported screening instruments (not clinical diagnostic measures) to measure mental health symptoms. This was achieved by examining (a) the prevalence of probable adjustment disorder at each wave; (b) trajectories of probable adjustment disorder over time; and (c) factors associated with the development of probable adjustment disorder 3–4 years after the fires, relative to no psychiatric disorder and probable other psychiatric disorder including PTSD and MDE.

## Method

### Participants and procedure

Eligible participants were at least 18 years of age and residents of 25 communities across 10 locations in Victoria at the time of the 2009 Black Saturday bushfires. The communities were selected because they were variably affected by the Black Saturday bushfires, ranging from a high level of impact (communities with fatalities and many houses lost), to medium impact (communities with few or no fatalities but significant property damage) and low impact (no fatalities, minimal or no property loss). To facilitate recruitment, the Victorian Electoral Commission provided contact details of adult current residents and those who had relocated since Black Saturday, totalling *n =* 7467 eligible participants. A personalised letter was sent to these adults, inviting them to participate in the study and including a reply-paid envelope to comply with local privacy laws. Participants provided written informed consent to participation. The procedure recruited a Wave 1 sample of *n* = 1056 residents, representing around 14% of eligible individuals. Wave 1 data collection occurred between December 2011 and January 2013, and at the completion of Wave 1, *n* = 966 participants agreed to be contacted at Wave 2. Wave 2 data collection occurred between July and November 2014 (5 years after the fires) and *n* = 736 participants took part, representing a retention rate of 76.1%. Wave 3 data collection occurred between April and August 2019 (10 years after the fires) and *n* = 524 participants took part, evidencing a retention rate of 49.6%.

Low-impact communities were characterised by no evidence of burning and were excluded from the current analyses to maximise the likelihood that the sample comprised individuals exposed to a traumatic stressor, as operationalised previously in studies using the same data-set.^31^ Thus, the sample for the current study consisted of individuals who lived in communities that were characterised by high or medium levels of fire-affectedness (as defined by evidence of at least two fatalities and significant property damage) and took part in Wave 1 (*n* = 802), Wave 2 (*n* = 596) or Wave 3 (*n* = 436) surveys. Of note, 369 of these participants completed all three waves of data collection, comprising the 10-year longitudinal sample for the current analyses.

The authors assert that all procedures contributing to this work comply with the ethical standards of the relevant national and institutional committees on human experimentation and with the Helsinki Declaration of 1975, as revised in 2008. All procedures involving human subjects/patients in the Beyond Bushfires study were approved by the University of Melbourne Human Research Ethics Committee (HREC1852721.1). At each wave of data collection, the self-report survey information was conducted via web-based or telephone format (as per participant preference).

### Measures

#### Probable adjustment disorder

Adjustment disorder is unique in its lack of specific, symptom diagnostic criteria,^34^ and is broadly described as emotional and/or behavioural symptoms that occur within 3 months following onset of a single psychosocial stressor, or ongoing psychosocial difficulties.^23^ In DSM-5, the central features of characterising these symptoms involve marked psychological distress and/or impairment in daily functioning that do not constitute another mental disorder.^23^ In the current study, probable DSM-5 adjustment disorder was assessed using the 6-item Kessler Psychological Distress Scale (K-6)^35^ and two supplementary items embedded within the K-6 that assess functional impairment. The K-6 is a well-validated measure of psychological distress (i.e. non-specific depression and anxiety symptoms) in the past 30 days, and comprises six items rated on a 5-point severity scale and five supplementary items assessing functional impairment and help-seeking.^36^ In the current study, a threshold of at least one psychological distress symptom *and* at least one total or half day of functional impairment in the absence of another psychiatric disorder (fire-related PTSD and/or MDE diagnosis as measured below) was adopted to reflect the probable occurrence of adjustment disorder. This method for estimating adjustment disorder is consistent with the method used in the O'Donnell et al (2016) study of traumatic injury survivors referred to above.^21^

#### Probable fire-related PTSD

Probable fire-related PTSD diagnosis was assessed using a four-item version of the Posttraumatic Stress Disorder Checklist (PCL-4).^37^ The four items, each scored on a 5-point severity scale, assess for intrusion, avoidance and arousal symptoms experienced in the previous 4 weeks. Adopting a cut-off score of seven achieves an efficient estimation of PTSD diagnosis relative to the full version of the PCL.^38^ To assess for fire-related PTSD, each PCL-4 item contained a follow-up question asking whether this PTSD symptom was related to the participant's reactions to the Black Saturday bushfires. The PCL-4 has demonstrated high levels of internal consistency and diagnostic utility.^39^

#### Probable depression

Probable MDE was assessed using the nine-item Patient Health Questionnaire (PHQ-9).^40^ The nine items correspond to the nine diagnostic criteria specified in DSM-5. A cut-score of five was adopted to reflect the probable occurrence of an MDE, indicating that at least five of the nine symptoms were experienced for most days or more often in the previous 2 weeks, consistent with prior analyses.^31–33^ The PHQ-9 has strong psychometric properties, with high levels of reliability and validity when used with comparable populations.^40^

#### Fire-related experiences

At Wave 1, the following questions were used to index the severity of direct exposure to the fires: (a) whether the participant had feared for their own life, (b) whether anyone close to the participant had died in the fires, and (c) whether they lost personal property or buildings in the fires.

#### Post-fire non-traumatic stressors

At Wave 1, participants were asked to indicate whether they had experienced any of the following major non-traumatic life stressors since exposure to the fires (i.e. in the past 3–4 years): adverse changes to their (a) income, (b) employment status, (c) occupation, (d) accommodation and (e) personal relationships. Among studies that have examined the impact of a set of disaster-related stressors on mental health outcomes, the most commonly utilised method involves analysing each stressor as a separate variable, regardless of *a priori* hierarchy.^14^ Consistent with this, each stressor in the current study was analysed as a separate predictor variable.

#### Post-fire traumatic stressors

At Wave 1, participants were asked to indicate whether they had been exposed to any of the following potentially traumatic events since exposure to the fires (i.e. in the past 3–4 years): (a) natural disaster, (b) serious accident and (c) serious assault/violence. These questions were asked to obtain an index of accumulated exposure to traumatic events following the fires.

### Statistical analyses

Data file preparation and preliminary analyses were conducted using SPSS Version 26 (for Windows). These involved estimation of prevalence rates for probable fire-related PTSD and MDE, which were calculated using frequency analysis at each wave. To depict the trajectories of probable adjustment disorder for the longitudinal sample (*n* = 369), prevalence rates (i.e. frequency data) of probable (PTSD and MDE) psychiatric diagnostic status across the three waves of data collection were plotted using the Sankey Diagram Generator (sankey-diagram-generator.acquireprocure.com). A Sankey diagram is a flow diagram that depicts transfers within a system, where the width of each band is proportional to the quantity being visualised (i.e. larger width represents a larger quantity).

A series of regression analyses were then conducted in subsequent stages of analyses, using MPlus Version 8 (for Windows). A series of multinomial logistic regression analyses were conducted first to explore whether probable adjustment disorder (relative to no (PTSD/MDE) psychiatric disorder) at Wave 1 increased risk for incident cases of probable adjustment disorder or PTSD/MDE psychiatric disorder (relative to no psychiatric disorder) at Wave 2 and Wave 3 respectively. These regression models controlled for demographic factors including gender, age at the time of the Black Saturday bushfires and highest level of education. To examine risk factors associated with the occurrence of probable adjustment disorder at Wave 1 (compared with no psychiatric disorder and probable (PTSD/MDE) psychiatric disorder), a multinomial logistic regression model was also estimated. Demographic variables, severity of fire exposure variables, and post-fire traumatic and non-traumatic stressors were all included as predictors. Multiple imputation techniques were used to manage missing data, with regression estimates aggregated across *k* = 20 imputed samples.

## Results

### Participant sociodemographics

Key sociodemographic characteristics for participants at each wave of data collection are presented in Table 1. The mean age of participants was 52.6 years at the time of the Black Saturday bushfires, and 62.8 years 10 years later (Wave 3). Across all waves, participants were predominantly Australian-born, women, non-tertiary educated, and current or past residents of communities with high levels of community fire-affectedness.

**Table 1:** Sociodemographic characteristics of participants

Characteristic	Wave 1 (*n =* 802)	Wave 2 (*n =* 596)	Wave 3 (*n =* 436)
Gender (female), *n* (%)	482 (60.1)	231 (38.8)	171 (39.2)
Age, years: mean (s.d.)	52.6 (12.3)	58.1 (12.7)	62.8 (12.7)
Australian-born, *n* (%)	673 (83.9)	498 (83.6)	365 (83.7)
Tertiary education, *n* (%)	296 (36.9)	242 (40.6)	192 (44.0)
Communities’ bushfire impact level, *n* (%)			
High impact^a^	667 (83.2)	504 (84.6)	376 (86.2)
Medium impact^b^	135 (16.8)	92 (15.4)	60 (13.8)

a. High impact: communities with fatalities and many houses lost.

b. Medium impact: communities with few or no fatalities but significant property damage.

### Prevalence of probable adjustment disorder

Frequencies of probable adjustment disorder, fire-related PTSD, MDE and no psychiatric disorder at each wave of data collection are presented in Table 2. At each wave, probable adjustment disorder was more prevalent than probable psychiatric disorder (fire-related PTSD, MDE). Rates of probable adjustment disorder remained relatively stable over time, although notably, the highest rate was estimated at Wave 3 (18.6%), which was 10 years after the fires.

**Table 2:** Prevalence of probable other psychiatric disorder

Probable psychiatric diagnostic status	Wave 1 (*n* = 802)		Wave 2 (*n =* 596)		Wave 3 (*n =* 436)	
	*n*	%	*n*	%	*n*	%
Adjustment disorder	126	15.7	88	14.8	81	18.6
Psychiatric disorder						
Fire-related PTSD	110	13.7	58	9.7	28	6.4
Depression	97	12.1	57	9.6	39	8.9
No psychiatric disorder	489	61.0	393	65.9	270	61.9

PTSD, post-traumatic stress disorder.

### Trajectory of probable adjustment disorder

**Fig. 1:**
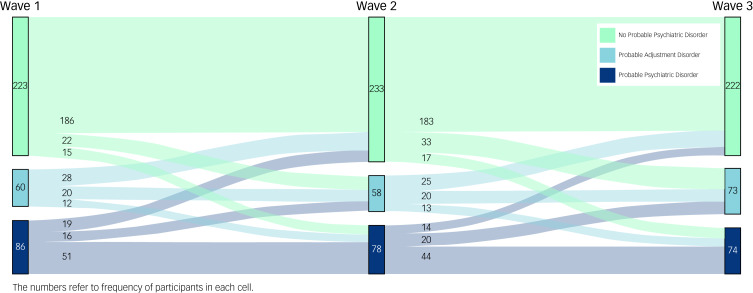
Sankey diagram showing the trajectory of probable other psychiatric diagnosis across the 10-year period following the Black Saturday bushfires in Australia.

Figure 1 demonstrates the trajectory of probable adjustment disorder over the 10 years following the Black Saturday bushfires, for the longitudinal sample (*n* = 369). This figure illustrates that there were considerable fluctuations between probable adjustment disorder and other probable diagnostic categories across time. By way of illustration, among participants with probable adjustment disorder at Wave 1, nearly half (47%) recovered, transitioning to no psychiatric disorder at Wave 2, while a considerable proportion (53%) either continued to have a probable diagnosis of adjustment disorder (33%) or escalated to a probable diagnosis of other psychiatric disorder (20%). A similar pattern emerged between Waves 2 and 3, where nearly half (43%) of those with probable adjustment disorder at Wave 2 recovered, transitioning to no psychiatric disorder, while the majority (57%) either maintained a diagnosis of probable adjustment disorder (35%) or escalated to probable other psychiatric disorder (22%). Across the 10-year post-disaster period, results demonstrate that approximately 1 in 3 participants with probable adjustment disorder continued to have probable adjustment disorder at the subsequent wave, and 1 in 5 participants with probable adjustment disorder escalated to a more severe psychiatric disorder (i.e. fire-related PTSD, MDE) at the subsequent wave.

Results from multinomial regression analysis examining the implications of probable adjustment disorder for incident cases of psychiatric disorder (controlling for gender, age and education) indicated that a classification of probable adjustment disorder (compared with no psychiatric disorder) at Wave 1 significantly increased risk for classifications of probable adjustment disorder (OR = 4.8, *P* < 0.001) and probable psychiatric disorder (OR = 5.7, *P* < 0.001) at Wave 2. Classification of probable adjustment disorder (compared with no psychiatric disorder) at Wave 1 also predicted significantly increased risk for probable adjustment disorder (OR = 4.3, *P* < 0.001) and probable psychiatric disorder (OR = 5.7, *P* < 0.001) at Wave 3.

### Predictors of probable adjustment disorder

In terms of risk factors associated with the experience of probable adjustment disorder at Wave 1, Table 3 presents the odds ratios (ORs) derived from multinomial regression analyses comparing indicators of probable adjustment disorder (which was the reference category) with no psychiatric disorder and probable psychiatric disorder. As shown, relative to no probable psychiatric disorder at Wave 1, probable adjustment disorder was associated with gender (OR = 1.61) and two post-fire stressors: negative changes in income (OR = 0.43) and negative changes in relationships (OR = 0.47). Taking the inverse of these estimates indicated that probable adjustment disorder (relative to no disorder) was more likely to be identified by women, and that reports of negative changes in income and relationships were associated with 2.3-fold and 2.1-fold increases in reports of probable adjustment disorder respectively. Further comparisons indicated that instances of probable psychiatric disorder (relative to probable adjustment disorder) were also more common among participants who reported post-fire relationship-related stressors (OR = 1.93), as well as those who reported fearing for one's life during the fires (OR = 1.90).

**Table 3:** Multinomial regression examining risk factors for probable adjustment disorder at Wave 1 (total *n* = 802)

Predictors	Odds ratio	95% CI	
		Lower	Upper
Probable adjustment disorder versus no psychiatric disorder			
Gender (male)	1.61*	1.04	2.49
Age	1.01	0.99	1.02
Educational level	1.08	0.71	1.64
Property loss	0.98	0.93	1.04
Fear for life in fire	0.75	0.49	1.16
Bereaved in fire	0.97	0.63	1.50
Traumatic events	0.71	0.45	1.11
Income stressor	0.43***	0.27	0.68
Employment stressor	0.79	0.47	1.34
Occupation stressor	0.88	0.47	1.67
Accommodation stressor	0.86	0.50	1.50
Relationship stressor	0.47*	0.24	0.93
Probable adjustment disorder versus probable other psychiatric disorder			
Gender (female)	1.22	0.73	2.04
Age	1.00	0.99	1.02
Educational level	0.85	0.52	1.38
Property loss	1.05	0.97	1.13
Fear for life in fire	1.90*	1.13	3.20
Bereaved in fire	1.61	0.99	2.61
Traumatic events	1.04	0.63	1.73
Income stressor	0.88	0.51	1.51
Employment stressor	1.02	0.56	1.85
Occupation stressor	1.50	0.77	2.91
Accommodation stressor	1.58	0.86	2.88
Relationship stressor	1.93*	1.01	3.69

*P < 0.05, ***P < 0.001.

## Discussion

To date, this exploratory study is the first to examine the prevalence, longitudinal trajectories and risk factors for probable adjustment disorder in the aftermath of exposure to a large-scale disaster. This large, longitudinal study contributes not only to a comprehensive understanding of adjustment disorder,^23^ but also to the disaster literature, which has predominantly focused on PTSD or MDE.^14^

### Adjustment disorder as a risk for more serious disorder

Consistent with previous research that found subthreshold symptoms to be more prevalent than psychiatric disorder in the immediate^11,12^ and long-term^13^ aftermath of disaster, the prevalence rate of probable adjustment disorder at each wave in the current study exceeded rates of probable fire-related PTSD and MDE. Differences in prevalence rates became most prominent 10 years after the fires, when the rate of probable adjustment disorder (18.6%) were approximately three times that of fire-related PTSD (6.4%) and double that of MDE (8.9%). Consideration of probable adjustment disorder in the present study points to the substantially increased mental health burden of disasters, relative to estimates based on prevalence of psychiatric disorders alone.

The Sankey diagram revealed that approximately one-third of those with a probable diagnosis of adjustment disorder at each wave continued to have a probable diagnosis at the subsequent wave. This rate is comparable to that in prior adjustment disorder research in a sample of traumatic injury survivors^21^ and emphasises that some people with adjustment disorder may experience persistent distress and impairment, which raises important questions regarding the diagnosis of adjustment disorder as a largely transient condition. Further, this finding underscores the importance of long-term assessment and monitoring of adjustment difficulties in populations exposed to community trauma and disasters.

Building on emerging evidence implicating adjustment disorder as a potential gateway to development of more severe disorders,^21^ results showed that probable diagnosis of adjustment disorder predicted a five-fold increase in risk for escalating to a diagnosis of more severe psychiatric disorder (i.e. incident cases of fire-related PTSD and/or MDE). In other words, for a subset of those with probable adjustment disorder, their symptoms accumulated and escalated over time, indicating that adjustment disorder may be an early marker for severe disorder. This finding accords with previous reports that subthreshold PTSD often escalates to PTSD over time^41^ and highlights that presence of subthreshold distress may be a useful marker to identify disaster survivors who could benefit from early intervention in order to disrupt this trajectory.

In delineating how adjustment disorder differs from more severe disorder, fearing for one's life during the Black Saturday bushfires emerged as a differential risk factor for probable (PTSD or MDE) psychiatric disorder. Consistent with research that has found fearing for one's safety to be a key predictor of PTSD,^38^ this finding highlights that fear may be a key factor distinguishing those who develop more severe disorder as opposed to adjustment difficulties following trauma.

### Risk factors for adjustment disorder

The current study found that stressors related to income and relationship changes were associated with an increased risk of having a probable diagnosis of adjustment disorder. This is largely consistent with post-disaster research linking the experience of ongoing financial, health and interpersonal stressors to negative long-term post-traumatic mental health outcomes.^27,28^ The conservation of resources (COR) theory^42^ provides a useful framework for understanding how these longer-term stressors may be contributing to post-disaster mental health. The COR theory conceptualises stressors as losses of personal, social and financial resources, and inherent to the theory is the tenet that persistent mental health symptoms are perpetuated by the process of ‘loss spirals’, whereby initial stressors and losses can lead to subsequent stressors in the same or different life domains.^42^ Moreover, the ‘loss spirals’ principle inherent to the COR theory suggests potential reciprocal relationships between stressors and post-disaster mental health, whereby either direction of causal effects between two variables of interest is plausible. When interpreted in light of current findings, this also points to the possibility that negative changes in income and relationships may operate as both a precipitant and a consequence of post-disaster mental health outcomes. This finding points to the important role of service providers in the provision of long-term support when responding to ongoing stressors in the disaster aftermath. Service responses that provide ongoing, long-term practical support to directly target post-disaster stressors, such as resources to assist with income difficulties and/or assisting with securing employment or permanent housing, have the potential to protect against mental health difficulties arising in the immediate and long-term aftermath of a disaster.

### Potential interventions for adjustment disorder

The identification of factors associated with the adjustment difficulties may facilitate the targeting of interventions to subclinical presentations. Given emerging evidence suggests that adjustment disorder sits on continuum of symptom severity between no disorder and severe psychiatric disorders,^21^ low-intensity interventions may be particularly useful for targeting adjustment difficulties following adversity. A number of low-intensity interventions are gaining an evidence base, particularly Problem Management Plus (PM+)^43^ and the Skills for Life Adjustment and Resilience program (SOLAR).^44–46^ Both of these interventions are brief transdiagnostic interventions that can be delivered by non-specialist laypeople in a task-shifting model, which is relevant to post-disaster contexts. SOLAR may be particularly useful because it is trauma-informed and has a module for addressing trauma memories. These low-intensity, brief transdiagnostic interventions may play a key role in mitigating the long-term course of adjustment difficulties in the wake of disaster.

### Limitations

Several limitations of the current study need to be acknowledged. The sample was restricted to participants from communities characterised as having suffered high and medium levels of bushfire impact (as defined by evidence of multiple fatalities and significant burning), and thus excluded participants classified as having experienced low impact (no evidence of burning). This is consistent with prior studies of the same data-set, which have used this area-based indicator to operationalise bushfire exposure,^31,47^ but does not account for individual variability in disaster experiences (including subjective experiences of threat). When this study was initiated, there was lack of brief instruments designed to assess DSM-5 criteria for adjustment disorder,^41^ and for consistency over time, the same measures were repeated. Consequently, a concise index of probable adjustment disorder was created using the K-6, and thus the probable diagnosis lacks reliability or validity information. The probable adjustment disorder index was centred on the clinical significance symptoms central to the diagnosis; however, there were limitations in the degree to which the probable diagnosis mapped onto remaining DSM-5 adjustment disorder diagnostic criteria. Specifically, we only excluded probable PTSD and MDE but not other psychiatric disorders (such as generalised anxiety disorder), which may overstate or complicate our estimates of probable adjustment disorder. Probable adjustment disorder and reports of post-fire stressors were both measured at Wave 1 (3–4 years after the fires), and there was no measure of pre-disaster mental health status. We were therefore unable to assess the temporal association of these variables and thus cannot determine at what point the onset of probable adjustment disorder occurred. Moreover, the causal associations between probable adjustment disorder and income and relationship stressors is unclear. This suggests that either direction of effects is plausible, highlighting the likely reciprocal relationships indicative of the described process of ‘loss spirals’ inherent to the COR theory. Last, the recruitment strategy resulted in only 14% of residents in the targeted regions participating in the study, potentially limiting the generalisability of the findings. These participants were more likely to be older, women and better educated (when compared with census data), with such characteristics also associated with varying rates of mental health problems over time.^31^

### Implications

This research speaks to the importance of symptoms that fall below diagnostic thresholds, into the subclinical space. Given our finding that those with probable adjustment disorder are at risk for later disorder, and are particularly vulnerable to financial and relationship stressors, it is essential that disaster responses include a focus on services for those with adjustment difficulties in addition to those with other psychiatric disorders.

## Data Availability

The data that support the findings of this study are available from the corresponding author, M.L.O., on reasonable request.
